# Comparison between data-driven clusters and models based on clinical features to predict outcomes in type 2 diabetes: nationwide observational study

**DOI:** 10.1007/s00125-021-05485-5

**Published:** 2021-05-31

**Authors:** Moa Lugner, Soffia Gudbjörnsdottir, Naveed Sattar, Ann-Marie Svensson, Mervete Miftaraj, Katarina Eeg-Olofsson, Björn Eliasson, Stefan Franzén

**Affiliations:** 1 Institute of Medicine, University of Gothenburg, Sahlgrenska University Hospital, Gothenburg, Sweden; 2 National Diabetes Register, Centre of Registers, Gothenburg, Sweden; 3 Institute of Cardiovascular and Medical Sciences, University of Glasgow, Glasgow, UK

**Keywords:** Cardiovascular diseases, Cluster analysis, Diabetes complications, Diabetes mellitus type 2, Epidemiology, Mortality

## Abstract

**Aims/hypothesis:**

Research using data-driven cluster analysis has proposed five novel subgroups of diabetes based on six measured variables in individuals with newly diagnosed diabetes. Our aim was (1) to validate the existence of differing clusters within type 2 diabetes, and (2) to compare the cluster method with an alternative strategy based on traditional methods to predict diabetes outcomes.

**Methods:**

We used data from the Swedish National Diabetes Register and included 114,231 individuals with newly diagnosed type 2 diabetes. *k*-means clustering was used to identify clusters based on nine continuous variables (age at diagnosis, HbA_1c_, BMI, systolic and diastolic BP, LDL- and HDL-cholesterol, triacylglycerol and eGFR). The elbow method was used to determine the optimal number of clusters and Cox regression models were used to evaluate mortality risk and risk of CVD events. The prediction models were compared using concordance statistics.

**Results:**

The elbow plot, with values of *k* ranging from 1 to 10, showed a smooth curve without any clear cut-off points, making the optimal value of *k* unclear. The appearance of the plot was very similar to the elbow plot made from a simulated dataset consisting only of one cluster. In prediction models for mortality, concordance was 0.63 (95% CI 0.63, 0.64) for two clusters, 0.66 (95% CI 0.65, 0.66) for four clusters, 0.77 (95% CI 0.76, 0.77) for the ordinary Cox model and 0.78 (95% CI 0.77, 0.78) for the Cox model with smoothing splines. In prediction models for CVD events, the concordance was 0.64 (95% CI 0.63, 0.65) for two clusters, 0.66 (95% CI 0.65, 0.67) for four clusters, 0.77 (95% CI 0.77, 0.78) for the ordinary Cox model and 0.78 (95% CI 0.77, 0.78) for the Cox model with splines for all variables.

**Conclusions/interpretation:**

This nationwide observational study found no evidence supporting the existence of a specific number of distinct clusters within type 2 diabetes. The results from this study suggest that a prediction model approach using simple clinical features to predict risk of diabetes complications would be more useful than a cluster sub-stratification.

**Graphical abstract:**

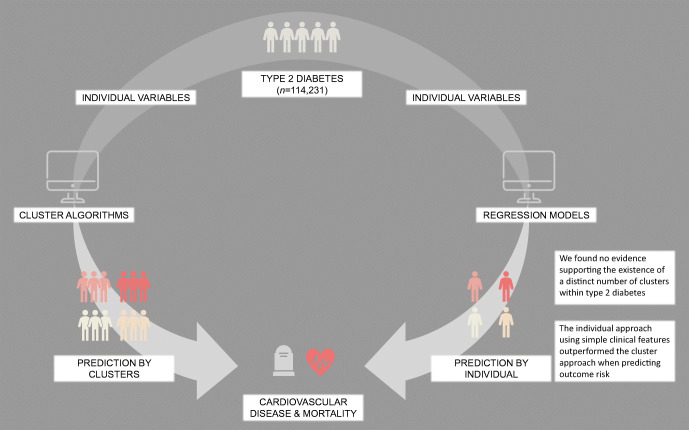

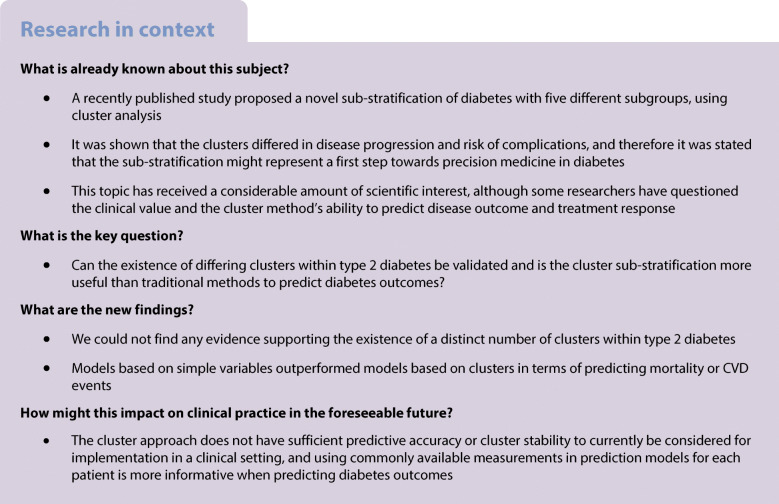



## Introduction

Type 2 diabetes is a highly heterogeneous disease in terms of clinical presentation, disease course and outcome. Both the expression and the progression of the disease are influenced by several different complex processes, making it difficult to predict prognosis and therapeutic response in affected individuals. A better characterisation and understanding of this heterogeneity may provide powerful tools to improve care and outcomes for patients, making this an area of interest for many researchers.

With the aim of providing a refined classification of diabetes, Ahlqvist et al. recently proposed five novel subgroups, using data-driven cluster analysis in the All New Diabetes in Scania (ANDIS) cohort [1]. The clusters were based on six measured variables: GAD autoantibodies (GADA), age at diagnosis, BMI, HbA_1c_ and HOMA estimates of beta cell function and insulin resistance. The first cluster was defined by the presence of GADA, therefore consisting of individuals with autoimmune diabetes (type 1 diabetes and latent autoimmune diabetes of adulthood [LADA]). Four type 2 diabetes clusters were then described based on the absence of GADA and varying degree of differences in the other mentioned variables. Using observational follow-up, Ahlqvist et al. were also able to show that the clusters differed in disease progression and risk of diabetes complications. The authors argued that the results represented a first step towards precision medicine in diabetes, and that sub-stratification may help to tailor and target early treatment to patients who would benefit the most.

The aforementioned study generated a great deal of scientific interest for this topic, and since it was published other researchers have applied the same clustering algorithm to different populations with results similar to the clusters originally proposed [2–6]. This suggests that the same clustering algorithm applied across different populations will produce similar clusters.

Even so, other researchers have questioned the value of this approach in terms of its utility for prediction of disease progression and therapeutic response [3, 7]. Dennis et al. used data from the ADOPT and RECORD trials and identified clusters that were similar to those described in the original study. They then compared the ability of a cluster approach to predict disease progression and treatment response with models based on simple continuous clinical features. The latter outperformed the cluster approach, leading the authors to the conclusion that the clinical relevance of the cluster approach is limited [3].

Cluster analysis is a complex field, and it is generally difficult to determine whether the clusters identified are in fact corresponding to a real underlying phenotypic grouping and are not just a result of dependency among variables. Cluster analysis is, in fact, dividing data objects into groups based only on information found within the data. The cluster algorithms will produce clusters wherever the variables used are correlated. Thus, the real challenge does not lie in creating clusters, but rather in demonstrating that the clusters created truly have captured a natural structure within the data, and also that they can add clinical value. In the present nationwide observational study using data from newly diagnosed individuals within the National Diabetes Register of Sweden (NDR), we aimed to find out if we could verify the existence of clusters of people with type 2 diabetes. To evaluate clinical utility, we compared the cluster method with an alternative strategy based on simple clinical features to predict cardiovascular events and mortality risk.

## Methods

### Study design

This study is a population-based cohort study of individuals in Sweden with newly diagnosed type 2 diabetes and aged 40 years or older, using data from the NDR and linking it to several national Swedish health registries through each unique Swedish personal identification number. We defined type 2 diabetes as diabetes in patients ≥40 years at diabetes diagnosis, treated with diet with or without oral glucose-lowering agents. Clinical characteristics at baseline and data on severe adverse events were obtained using NDR, the Swedish National Patient Register and the cause of death register.

The Regional Ethical Review Board of the University of Gothenburg approved the study which conformed with the Helsinki Declaration of 1964, as revised in 2013, concerning human and animal rights.

NDR has been described previously [8]. This register includes information on risk factors, complications of diabetes, and medications for patients aged 18 or older. Each patient provides consent for inclusion in the register, and virtually all individuals in Sweden with diabetes are included [9]. The Swedish National Patient Register contains nationwide hospital discharge information, diagnoses and procedures from all specialist care (in-hospital and outpatient); data on ICD-10 codes, procedure codes and date of contact. Diagnoses in the Swedish National Patient Register are registered according to the International Classification of Diseases (ICD) 9th and 10th revision. The cause of death register contains nationwide cause of death data based on death certificates, classified according to ICD-10.

In this study we defined CVD events as first post-index occurrence of any of the following diagnoses captured by the patient register: ischaemic heart disease (ICD codes I20–I25), cardiac arrest (ICD codes I46.0, I46.1 and I46.9), intracerebral haemorrhage (ICD code I61), cerebral infarction (ICD code I63) or stroke, not specified as haemorrhage or infarction (ICD code I64).

### Statistical methods

Means and SDs are presented for continuous variables, and percentages for categorical variables. Missing data were imputed using a single stochastic imputation from a multiple chained equation procedure [10].

The cluster analysis is based on the *k*-means algorithm using imputed and normalised observations on age, BMI, HbA_1c_, systolic BP, diastolic BP, HDL-cholesterol, LDL-cholesterol, triacylglycerol and kidney function (eGFR) from individuals with newly diagnosed type 2 diabetes [11, 12]. The *k*-means algorithm is based on the Euclidean distance and is therefore sensitive to differences in scales between variables, and so the data were normalised prior to the cluster analysis. The normalisation was made by subtracting the variable mean from each observation and then dividing it by the variable SD ensuring that all variables have mean zero and SD one. The elbow method was used to search for optimal number of clusters evaluating up to ten clusters. The elbow method is a heuristic method that uses the relation between cluster variability, measured by the within-cluster sum of squares, to support the identification of an optimal number of clusters [13]. Essentially, the *k*-means cluster analysis is performed multiple times with different values of *k*, and the within-cluster sum of squares is calculated and plotted for each *k*. The optimal number of clusters is inferred as a point on the elbow plot beyond which there is only a small reduction on the within-cluster variability, visually represented as the ‘bend’ of the elbow.

To get a reference for what the elbow plot would look like in the absence of any clusters, we estimated the covariance matrix for the normalised NDR data and simulated data from a normal distribution with the estimated covariance matrix. These simulated data have the same variance–covariance structure as original real data but consist only of one cluster. We evaluated up to ten clusters on the simulated data, which gave a hint of what the elbow plot would look like in the absence of any clusters.

Three additional methods were used to assess the optimal number of clusters on a sampled dataset consisting of 20,000 observations. The silhouette method evaluates how well each object lies within its cluster and can be used to estimate the mean distance between clusters [14]. The silhouette coefficients range from −1 to +1, where a high value indicates that the object is well matched to its own cluster and poorly matched to neighbouring clusters. A negative value indicates that the object might have been assigned to the wrong cluster. The gap statistics compare the total intra-cluster variation between observed data and reference data with a random uniform distribution (a distribution with no obvious clustering) for different values of *k* [15]. The optimal value of *k* is interpreted as the one that maximises the gap, meaning that the clustering structure is far away from the uniform null distribution. In this analysis, 50 bootstrap samples were used. The Hopkins statistic measures the clustering tendency of a dataset by comparing the distance between each point and its nearest neighbour in the observed data with the distance to the nearest point in a simulated uniformly distributed dataset [15]. Values close to zero indicated no cluster tendency and values close to one indicated a strong clustering tendency.

Cluster models with up to seven clusters were evaluated as categorical predictors of mortality and incidence of CVD using Cox proportional hazard models. The cluster-based predictor models were benchmarked against two different Cox models that used all the nine underlying variables as independent variables. The first one was a simple proportional hazards model where each of the variables were included as main effect only. The second one was a more complex model that used smoothing splines with three *df* for each variable. This makes it more flexible since the splines allow for potential nonlinear effects of each variable.

The prediction models were compared using concordance, defined as the proportion of pairs of individuals in the data where the model and the observed outcome are concordant [16]. In other words, the person in the pair with the lower risk experiences the observed outcome later than the person with the higher risk score. A value of 1 represents perfect concordance.

The statistical analysis was performed using R 4.0.2 (CRAN https://cran.r-project.org/) and specifically the mice package (version 3.33.0) for imputation, the cluster (version 2.1.0), NBclust (version 3.0) and factoextra (version 1.0.7) packages for the cluster analysis.

## Results

This study included 114,231 individuals from the NDR, and baseline characteristics of the population are given in Table 1. The mean age was 62.8 years and 43.1% of the individuals were women. Mean BMI was 30.5 kg/m^2^ and the mean HbA_1c_ was 54.3 mmol/mol (7.4%). HDL-cholesterol, LDL-cholesterol and triacylglycerol was 1.2, 3.1 and 2.0 mmol/l, respectively. Mean eGFR was 84.8 ml min^−1^ [1.73 m]^−2^ and mean systolic and diastolic BP was 137.2 mmHg and 79.6 mmHg, respectively. The median follow-up time was 5.2 years with IQR from 3.7 to 7.3.

**Table 1:** Descriptive statistics for the total population and for four clusters

Variable	Total population (*N* = 114,231)	Cluster 1 (*n* = 12,133)	Cluster 2 (*n* = 27,888)	Cluster 3 (*n* = 41,555)	Cluster 4 (*n* = 32,825)
Sex, female	43.1	32.0	44.5	49.5	38.0
Age at diagnosis, years	62.8 ± 12.78	57.0 ± 11.68	65.4 ± 9.70	71.5 ± 9.01	51.8 ± 9.99
BMI, kg/m^2^	30.5 ± 5.65	31.6 ± 5.97	30.8 ± 5.19	28.0 ± 4.26	32.9 ± 6.17
HbA_1c_, mmol/mol	54.3 ± 17.10	89.5 ± 21.12	50.9 ± 10.88	49.0 ± 10.09	50.9 ± 10.48
HbA_1c_, %	7.1	10.3	6.8	6.6	6.8
Systolic BP, mmHg	137.2 ± 17.37	137.8 ± 15.98	155.8 ± 14.88	132.0 ± 13.07	127.9 ± 11.69
Diastolic BP, mmHg	79.6 ± 10.04	82.9 ± 9.60	88.6 ± 8.20	73.3 ± 7.69	78.7 ± 7.82
Triacylglycerol, mmol/l	2.0 ± 1.33	3.8 ± 2.55	1.8 ± 0.90	1.6 ± 0.78	2.0 ± 0.98
HDL-cholesterol, mmol/l	1.2 ± 0.38	1.0 ± 0.28	1.3 ± 0.35	1.4 ± 0.43	1.1 ± 0.27
LDL-cholesterol, mmol/l	3.1 ± 1.00	3.5 ± 1.08	3.4 ± 0.99	2.8 ± 0.92	3.1 ± 0.94
eGFR, ml min^−1^ [1.73 m]^−2^	84.8 ± 24.57	97.1 ± 26.99	80.7 ± 19.85	71.9 ± 18.35	100.0 ± 23.69
Country of birth					
Sweden	80.1	77.1	72.5	82.9	85.5
Nordic countries (excl. Sweden)	5.3	4.9	4.4	6.3	5.5
Europe (excl. EU27 & Nordic countries)	3.0	3.7	4.0	2.9	2.2
EU27 (excl. Nordic countries)	2.5	2.4	2.3	2.7	2.7
Mediterranean countries	0.4	0.5	0.5	0.4	0.4
Middle East	4.5	5.6	8.8	2.7	1.9
Asia	1.5	2.2	2.9	0.6	0.7
South America	0.7	1.0	1.1	0.5	0.4
North America & Oceania	0.2	0.3	0.4	0.2	0.1
Africa	1.6	2.5	3.0	0.8	0.5

Data are means ± SD or percentages

Clinical characteristics for the NDR population as a total and divided into four clusters

The cluster analysis is based on the *k*-means algorithm using imputed and normalised observations on age, BMI, HbA_1c_, systolic BP, diastolic BP, triacylglycerol, HDL-cholesterol, LDL-cholesterol and eGFR from individuals with newly diagnosed type 2 diabetes

The regions represent countries of birth grouped in larger areas

**Fig. 1:**
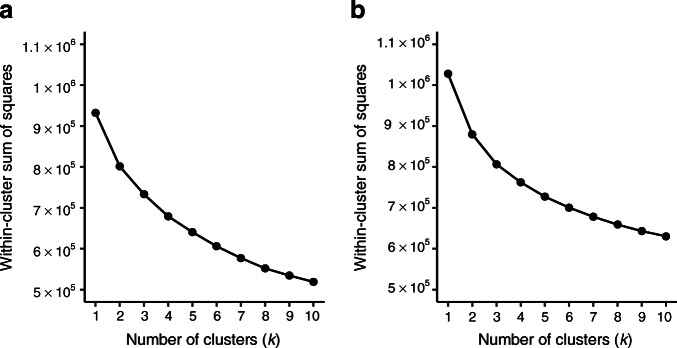
Elbow plot for real dataset (**a**) and elbow plot for simulated dataset (**b**). (**a**) The elbow plot is made by calculating the within-cluster sum of squares with *k* ranging from 1 to 10 in the NDR population. (**b**) A multivariate normal distribution is fitted to the data (the same data as used for the clustering), producing a vector of estimated averages and a covariance matrix. The estimated averages and covariance matrix are then used to simulate a dataset of the same size and structure as the data used for the real cluster analysis, except that the simulated data only have one cluster. The simulated data are then subjected to the same *k*-means clustering algorithm as the observed data

The elbow method was performed by calculating the within-cluster sum of squares with *k* ranging from 1 to 10 to visualise the optimal number of clusters for the studied population (Fig. 1a). The elbow plot showed a smooth curve without any clear cut-off points, making the optimal value of *k* unclear. A simulated dataset was created consisting only of one cluster, and an elbow plot was made using the same technique as for the real dataset (Fig. 1b). This elbow plot had a similar appearance as the one made with the real dataset, with a smooth curve and no clear cut-off points.

**Fig. 2:**
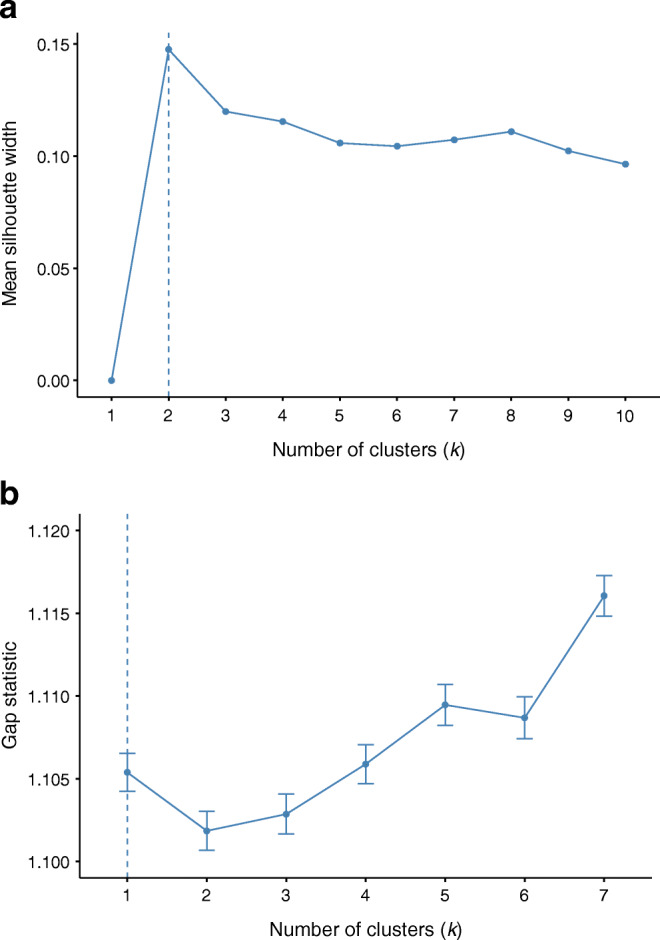
Silhouette method on sampled dataset (**a**) and Gap statistics on sampled dataset (**b**). (**a**) The silhouette method was performed on a sampled dataset consisting of 20,000 observations. The vertical dashed line indicates that the mean silhouette width was highest for *k* equal to 2 in this study. The Silhouette method is used to evaluate how well each person lies within their cluster and to estimate the mean distance between clusters. The silhouette coefficients range from −1 to +1, where a high value indicates that the individuals are well matched to their own clusters and poorly matched to neighbouring clusters. (**b**) Gap statistics were performed on a sampled dataset consisting of 20,000 observations, using 50 bootstrap samples. The gap statistic compares the total intra-cluster variation between observed data and reference data with a random uniform distribution (a distribution with no obvious clustering) for different values of *k*, the number of clusters. The optimal value of *k* is interpreted as the one that maximises the gap, in the figure indicated by the vertical dashed line

Using the silhouette method with values of *k* ranging from 1 to 10, the mean silhouette width was highest for *k* equal to 2. However, for all values of *k* the silhouette score was close to 0, ranging from 0.00–0.15 (Fig. 2a). When using the gap statistics, there was only a slight hint towards 1 being the optimal value of *k* (Fig. 2b). The Hopkins statistic for clustering tendency was 0.15.

**Fig. 3:**
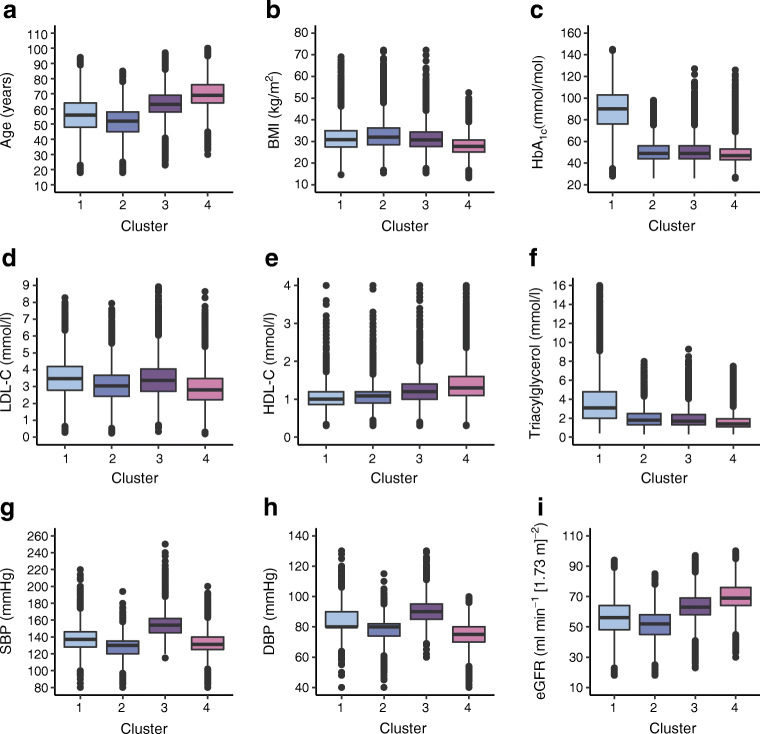
Cluster characteristics in four clusters. (**a**–**i**) Distribution of (**a**) age at diagnosis, (**b**) BMI, (**c**) HbA_1c_, (**d**) LDL-C (LDL-cholesterol), (**e**) HDL-C (HDL-cholesterol), (**f**) triacylglycerol, (**g**) SBP (systolic BP), (**h**) DBP (diastolic BP) and (**i**) eGFR at baseline for each of the four clusters

Given the failure to identify a specific number of clusters, we nevertheless proceeded to investigate modes with up to five clusters. The baseline characteristics for four clusters are described using standard descriptive statistics for the nine continuous variables used in the analysis (Table 1). Cluster 1 was characterised by a relatively low age at diagnosis, high HbA_1c_, relatively high BMI, BP, LDL-cholesterol and triacylglycerol, and low HDL-cholesterol. Individuals in cluster 2 were older at diagnosis and had lower HbA_1c_ levels and BMI. They had the highest BP of all subgroups. The age at diagnosis was highest in cluster 3, and they had the lowest levels of HbA_1c_ and BMI. They also had low BP, LDL-cholesterol and triacylglycerol levels. This was also the cluster with the lowest eGFR levels. Cluster 4 were the youngest at diagnosis and had the highest BMI of the subgroups. Their HbA_1c_ levels were high, and their eGFR levels were the highest of all groups (Fig. 3).

When comparing our clusters to the ones described in the ANDIS cohort, cluster 4 is most comparable with MOD (mild obesity-related diabetes) since it has the highest BMI and low age at diagnosis [1]. Cluster 3 resembles MARD (mild age-related diabetes) with the highest age at diagnosis and low BMI and HbA_1c_ levels. Cluster 1 is closest to SIDD (severe insulin-deficient diabetes) with the highest HbA_1c_ levels and relatively low age at diagnosis. Cluster 2 and SIRD (severe insulin-resistant diabetes) are fairly well matched since they both have relatively high age at diagnosis and low HbA_1c_ levels.

To evaluate the ability to predict mortality risk and CVD events, comparisons were made for the different numbers of clusters using Cox regression models. Concordance was calculated for Cox models based on 2–7 clusters, as well as a Cox model with the variables as they are and a Cox model with splines for all variables. In prediction models for mortality, concordance was 0.63 (95% CI 0.63, 0.64) for two clusters, 0.66 (95% CI 0.65, 0.66) for four clusters, 0.77 (95% CI 0.76, 0.77) for the ordinary Cox model and 0.78 (95% CI 0.77, 0.78) for the Cox model with splines for all variables. In prediction models for CVD events, the concordance was 0.64 (95% CI 0.63, 0.65) for two clusters, 0.66 (95% CI 0.65, 0.67) for four clusters, 0.77 (95% CI 0.77, 0.78) for the ordinary Cox model and 0.78 (95% CI 0.77, 0.78) for the Cox model with splines for all variables (Table 2).

**Table 2:** Concordance for prediction models for mortality and CVD events using 2–7 clusters, an ordinary Cox model and a Cox model with smoothing splines

No. of clusters/model	Mortality		CVD	
	Concordance	95% CI	Concordance	95% CI
2 cluster	0.63	0.63, 0.64	0.64	0.63, 0.65
3 cluster	0.63	0.63, 0.64	0.64	0.63, 0.65
4 cluster	0.66	0.65, 0.66	0.66	0.65, 0.67
5 cluster	0.66	0.65, 0.66	0.68	0.67, 0.69
6 cluster	0.65	0.65, 0.66	0.68	0.67, 0.68
7 cluster	0.66	0.66, 0.66	0.68	0.67, 0.69
PH Cox	0.77	0.76, 0.77	0.77	0.77, 0.78
Spline Cox	0.78	0.77, 0.78	0.78	0.77, 0.78

PH Cox, Cox model with the variables as they are

Spline Cox, smoothing splines for all variables

## Discussion

In this nationwide study, using data from 114,231 individuals in the NDR, we found no robust evidence supporting the existence of a specific number of clusters within type 2 diabetes based on the use of common clinical variables. The fact that we were unable to establish an optimal number of clusters within the studied population implies that the cluster division may be arbitrary. This is further illustrated by the similarity of the different elbow plots, where one is made from the real dataset and one is made from a simulated dataset with only one cluster. The smooth appearance of the plots is to be expected in the simulated data since it does not contain any clusters, but we should see a marked difference in the plot from the real data, if it did in fact consist of separate conceptually meaningful groups. We then created a model with four clusters, to match the number of clusters with participants with type 2 diabetes in the ANDIS cohort, since the fifth cluster in the ANDIS cohort consisted of patients with type 1 diabetes and LADA [1]. The four clusters created in our study were sufficiently alike to assign similar cluster labels to those used in the original study. This is to be expected since the variables are dependent on each other and therefore will relate to one another in a predictable way across different populations. What we were able to show with the elbow plot is that the cluster quality is not markedly better when using four clusters than when using any other number of clusters. This contradicts the belief that the clusters, at least those derived from commonly available clinical characteristics worldwide, represent different diseases with separate underlying aetiologies that make them naturally fall into separate compartments.

A recently published study used data from the DEVOTE, LEADER and SUSTAIN trials and assigned individuals based on Euclidean distance to nearest cluster centre instead of a de novo cluster analysis. To evaluate the accuracy of the cluster assignment, a ratio between the smallest and the second smallest Euclidean distance to the ANDIS cluster centres was calculated. Across all three trial cohorts, this ratio showed a weak patient–cluster association with numbers closer to one than to zero [5]. However, the only variables used to form the clusters in this study were HbA_1c_, age at diagnosis and BMI, which might have influenced the result of the cluster replication. Still, this can be used to illustrate one important deficit of the clustering approach. Many individuals will have almost the same chance of being allocated into several clusters and will therefore be forced into one cluster even though the probability of being assigned to a different cluster is only marginally smaller.

A question raised in the original article on clusters in diabetes was if patients can move between clusters as the disease progresses [1]. A recent study performed cluster assignment according to distance to the nearest cluster centre at baseline and then again after 5 years and found that 23% of the participants had changed cluster after 5 years’ disease duration [6]. This is natural, since the cluster allocation is based on variables that can change over time, making the cluster assignment time dependent.

With the aim of evaluating the clinical utility of the cluster approach, we calculated and compared concordance for different prediction models and found that models based on simple variables outperformed models based on clusters in terms of predicting mortality risk or CVD events. This was independent of numbers of clusters, although the concordance increased slightly as the numbers of clusters increased. This has been demonstrated previously by Dennis et al., who for instance showed that even though the incidence of chronic kidney disease differed between clusters, eGFR at baseline was a better predictor of time to chronic kidney disease [3]. This can be explained by the fact that clustering leads to loss of information. The same variables that are used to assign individuals into broad clusters could instead be used as the exact value to predict outcome and therapeutic response for each individual, making the care more personalised and precise. The multifaceted pathophysiology of type 2 diabetes integrates many aspects of lifestyle, genes, environment and potentially programming. Each person with diabetes has different contributions from each with further changes across the diabetes life course [17]. Hence, it might be overly optimistic to assume that participants can be easily demarcated into a small number of distinct clusters that would add clinical precision and value.

A major strength of the present study is its large population size with nationwide scope with almost complete national coverage. All included variables in the analysis are easily measured and a common part of the clinical routine. In the original article from ANDIS, hierarchical clustering was used as part of the clustering analyses [1]. Owing to the large dataset in the present study (*n* = 114,231), this approach was not a feasible choice as it requires an excess of 200 GB of memory and therefore *k*-means clustering alone was used. For the same reason, the elbow method was considered to be the best choice to use when searching for the optimal number of clusters. Alternative methods such as the silhouette method and gap statistics are based on a distance matrix with all pairwise distances which, for the full dataset, are extremely computationally intensive. However, the silhouette method, the gap statistics and the Hopkins statistics were performed on a subset of the data (*n* = 20,000). The silhouette method and the gap statistics, like the elbow method, failed to provide conclusive evidence for an optimal number of clusters. The result of Hopkins statistics that assess the clustering tendency was 0.15 for our dataset. Generally, when this value is below 0.5 it is considered unlikely that the dataset contains statistically significant clusters [18].

The clinical variables used in the cluster algorithm differed between the original study and the present study, since Ahlqvist et al. included homoeostatic model assessment of two estimates of beta cell function and insulin resistance that were not available in our dataset. This is a limitation of the present study since insulin production and insulin sensitivity are indeed important mechanisms in the complex pathogenesis of diabetes [19]. However, these measurements are not available in the vast majority of clinical datasets worldwide. Since C-peptide is not routinely obtained, and also varies considerably between different laboratories, it may not yet be an optimal variable to include in clinical prediction models [20]. Although we did not have access to all of the variables used in the original study, we did have access to several other useful measurements such as eGFR, BP, HDL-cholesterol, LDL-cholesterol and triacylglycerol, contributing to the accuracy of our model, and the definition of type 2 diabetes we used most likely will exclude almost all individuals with type 1 diabetes or LADA. The differences in variables do not seem to have affected the analyses in a significant way, since the four clusters produced in our study were broadly similar to the originally proposed clusters. Another limitation of the current study is missing data. Missing data are unfortunately common in NDR, as in most clinical observational datasets. To deal with this, a single stochastic imputation was used since ‘missing at random’ was considered more realistic than ‘missing completely at random’ in the dataset [21].

The choice to use concordance to evaluate the prediction models was mainly driven by its relatively intuitive nature that makes it easy to explain and understand. The main alternative would be to use Heller’s R^2^, but the way the SE for the estimated R^2^ is defined makes it difficult to calculate confidence intervals when used for penalised Cox models with smoothing splines.

The study from ANDIS was indeed important and meaningful, pointing out the need for further individualisation and precision medicine in diabetes. It also highlighted the fact that a lot of the knowledge that we have already accumulated regarding risk factors, differences in disease progression and therapeutic response has not yet been translated into clinical practice. How to approach this gap between knowledge and practice remains to be seen, and the future of precision medicine in diabetes might very well include diagnostic algorithms to define diabetes subtypes to guide therapeutic managements, as predicted by EASD/ADA [22]. While we fail to identify a specific number of clusters, that does not, however, mean that there could be no true underlying clusters, but the results from our study indicate that the cluster approach does not have sufficient predictive accuracy or stability to currently be considered for implementation in a clinical setting.

### Conclusion

In conclusion, using routinely available data in clinical practice, we could not find evidence supporting the existence of a distinct number of clusters within type 2 diabetes. Furthermore, the results from this study suggest that an alternative approach using simple clinical features to predict risk of diabetes complications is more useful than a sub-stratification approach.

## Data Availability

All data generated or analysed during this study are included in this published article.

## Abbreviations

ANDIS: All New Diabetes in Scania
GADA: GAD autoantibodies
LADA: Latent autoimmune diabetes of adulthood
NDR: National Diabetes Register of Sweden
